# Editorial: Stress response signaling in tumor development and its implications for cancer treatment

**DOI:** 10.3389/fonc.2022.1010222

**Published:** 2022-09-01

**Authors:** Danwan Wen, Peng Zhao

**Affiliations:** ^1^ Department of Biochemistry and Structural Biology, University of Texas Health Science Center at San Antonio, San Antonio, TX, United States; ^2^ Department of Radiation Oncology, The First Affiliated Hospital of Sun Yat-sen University, Guangzhou, China; ^3^ Mays Cancer Center, University of Texas Health Science Center at San Antonio, San Antonio, TX, United States

**Keywords:** stress response, cancers, therapeutic target and strategy, cancer diagnosis, signaling transduction

Organisms are inevitably exposed to environmental stress during daily life. Homeostasis maintaining programs, such as unfolded protein response, oxidative stress response, and integrated stress response, help restore homeostasis or adapt to the stress. However, the severe and prolonged stress or dysregulated stress response could cause tissue injury, metabolic dysfunction, inflammation, and even cancer. Therefore, dysregulated stress response is closely related to the incidence and progression of cancer. During tumorigenesis, genomic mutations and alterations in gene expression and protein functions could influence stress response, and thus promote cancer progression and metastasis. On the other hand, cancer treatments cause stress in cancer cells to induce cell death. Therefore, stress response within cancer cells substantially affects the efficacy of therapeutics. In this Research Topic, mechanisms of dysregulated stress response signaling pathways leading to cancer and involving in cancer treatment are explored to shed light on cancer development, progression, treatment as well as follow-up management (**Figure 1**).

**Figure 1:**
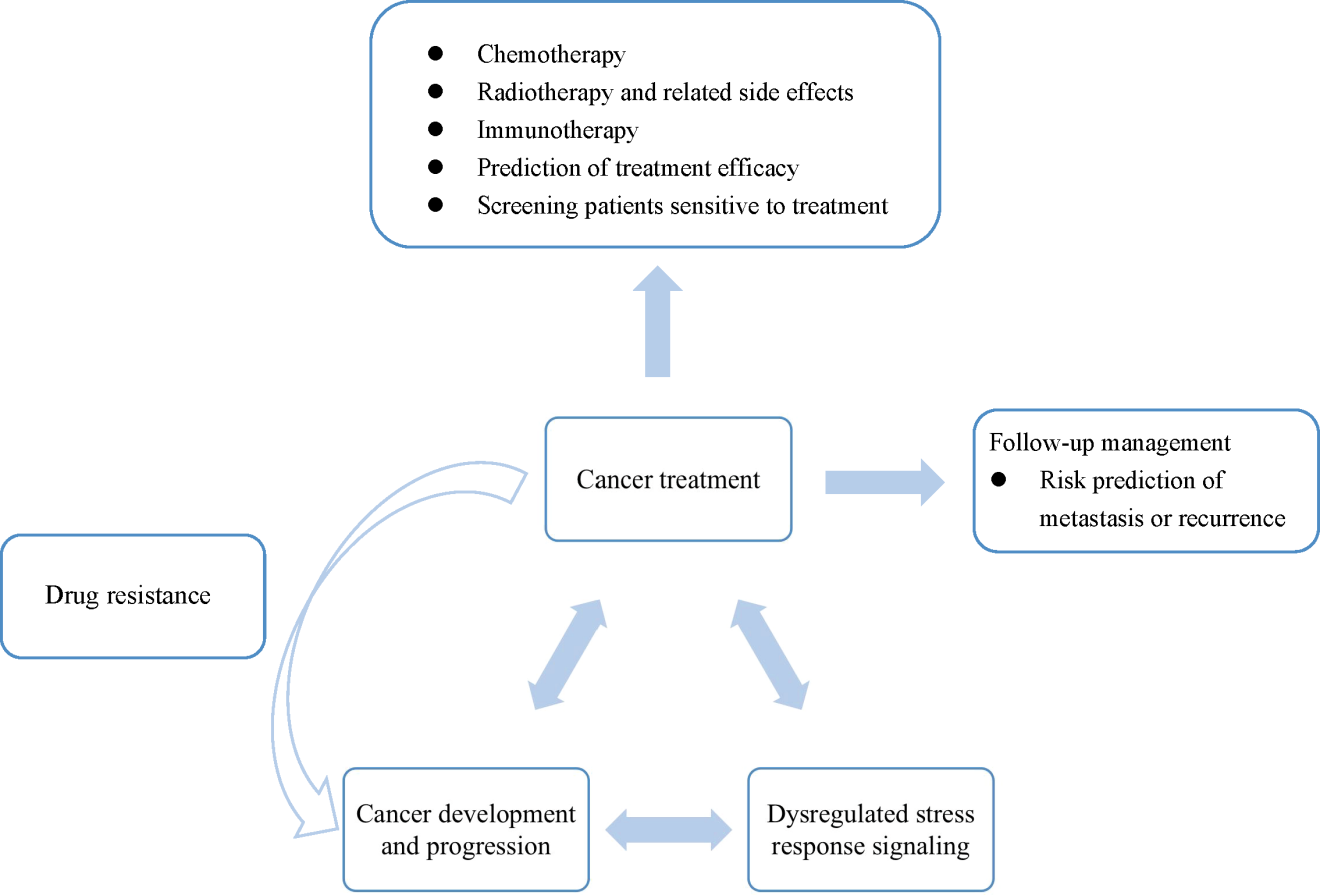
Understanding the interaction among dysregulated stress response, cancer development and progression, and cancer treatment benefits cancer diagnosis, therapeutics development, and post-treatment management.

## Dysregulated stress response in the initiation and progression of cancer

Dysregulated stress response is closely associated with cancer development and progression. The studies reported in this special issue further investigated molecular mechanism that underlies stress response during tumorigenesis. Tao et al. indicated that as a molecular link between persistent endoplasmic reticulum (ER) stress and metabolism, ER protein disulfide isomerase-associated 2 regulates metabolic reprogramming and mitigate cancerous transformation of chronic colitis. Su et al. reported that DNA polymerase Iota could induce Erk-O-GlcNAc transferase to excessively activate glucose-6-phosphate dehydrogenase, thus promoting the proliferation and progression of esophageal squamous cell carcinoma. Besides, long non-coding RNA (lncRNA) BCAS1-4_1 and H19 were shown to upregulate ZEB1 and then enhance the proliferation and metastasis of ovarian cancer cells and glioma cells (Xue et al., Chen et al.). Additionally, inositol requiring enzyme 1 alpha (IRE1α), a key regulator of unfolded protein response, was reported to enhance the progression of castration-resistant prostate cancer cells by a positive feedback loop of IRE1α/IL-6/androgen receptor pathway (Yang et al.).

With the emergence of COVID-19, patients with cancer may suffer from double stress from COVID-19 and cancer. In this issue, researchers tried to review and figure out the connection between COVID-19 and cancer *via* dysregulated stress response signaling, with the goal to alleviate their mutual impacts. They found that COVID-19 was associated with lung cancer and head and neck cancer *via* reactive oxygen species (ROS) signaling pathway and renin angiotensin system axis (Zhu et al., Iftikhar et al.).

Moreover, stress granules (SGs) play a critical role in the stress response, as they could reduce stress-related damage and improve cell survival through regulating translation, mRNA stability, and ability to work in cytoplasm and nucleus. Two detailed reviews summarized that SGs took part in initiation, progression and metastasis of cancer, and functioned in the mechanism of action of anti-cancer medications, such as sorafenib and 5-fluorouracil (Asadi et al., Asadi et al.).

## Cancer treatment

Cancer treatment methods include surgery, chemotherapy and radiotherapy, as well as immunotherapy and targeted therapy. These treatments could induce stress not only to cancer cells, but also to normal tissue cells. Stress response signaling could affect the treatment efficacy, with clinical implications for prognosis prediction and individualized treatment.

Different chemotherapy drugs could exert anticancer effects by affecting different stress response signaling pathways. It was reported that valproic acid and hydroxyurea could regulate homologous recombination and cell cycle through an MUS81-pRPA2 pathway in a synergistic manner to reduce breast cancer cells (Su et al.). Costunolide could activate apoptosis and autophagy of gastric cancer by promoting ROS pathway and meanwhile inhibiting AKT/GSK3β pathway (Xu et al.). Besides, cardiac glycoside ouabain could effectively reduce the expression and phosphorylation of STAT3 to inhibit the growth of cancer cells, such as non-small cell lung cancer (NSCLC) (Du et al.).

Radiotherapy is one of the most precise cancer treatments. For better therapeutic efficacy, researchers tried to enhance radiation sensitivity. Disulfiram could be applied as a radiosensitizer on pancreatic cancer treatment both *in vitro* and *in vivo* (Xu et al.). In addition, lncRNA TLCD2-1 was associated with radiation resistance and growth of cancer cell, suggesting that it could be targeted to improve radiation sensitivity and used as a prognostic biomarker in colorectal cancer (Yu et al.). Feng et al. summarized that 5,6,7,8-Tetrahydrobiopterin (BH4)/nitric oxide synthases (NOS) axis is a double edge sword in the regulation of radioactive sensitivity, because BH4 could lead to the generation of NOS and large oxidative free radicals after ionizing radiation, while promoting vascular normalization and thus enhancing radiotherapy efficacy.

Although the number of cancer cells could be reduced by radiotherapy, normal tissue cells also suffer from radioactive injury meanwhile. Therefore, the radiation-induced side effects can’t be ignored. It is important to understand the mechanisms of the incidence and treatment of complication. It was believed that over-activating radiation-induced immunological side effects could be harmful, as it could dramatically increase the secretion of pro-inflammatory cytokines, although moderate immune activation could be beneficial for cancer treatment (Zhang et al.). Besides, Xue et al. demonstrated that NF-E2-related factor 2 ameliorates the radiation-induced skin injury by regulating ROS pathway. Li et al. used network pharmacological analysis to identify the possible target genes and signaling pathways that underlies how radix salvia miltiorrhizae relieves radiation-induced pneumonia. Moreover, Chen et al. reviewed the physical classification, basic pathogenesis, clinical characteristics, predictive and diagnostic factors, and possible treatment targets of radiotherapy-induced digestive injury, and pointed out that more precise radiotherapy plan and gut microbiota modulation may help improve radiogenic gastrointestinal syndrome.

In addition to chemotherapy and radiotherapy, immunotherapy is an emerging and effective cancer treatment that significantly improves survival and life quality of patients. However, not all patients are sensitive to immunotherapy. Therefore, it is great importance to identify patients who may be beneficial from immunotherapy and develop individualized treatments. Sun et al. found that smoking patients of NSCLC had stronger immunogenicity and more activated immune microenvironment than non-smoking ones, so that immune checkpoint inhibitors may be more suitable for smoking patients of NSCLC. Xie et al. reported that Titin mutation was related to higher tumor mutation burden and better antitumor immune response in lung squamous cell carcinoma.

## Post-treatment management of cancers

After cancer treatment, proper follow-up management of patients can help recognize tumor metastasis or recurrence immediately, so that corresponding treatment measures can be taken to improve prognosis. Risk prediction of metastasis or recurrence could provide valuable reference for clinical individualized treatments. Chen et al. explored risk factors on the incidence and prognosis of colorectal cancer with brain metastasis (CRCBM). They found that the positive level of CEA, pN2a-b, and distant metastases were risk factors for the incidence of CRCBM, while systematic treatment was related to better survival of CRCBM patients.

## Hepatocellular carcinoma

Among various kinds of cancers, hepatocellular carcinoma (HCC), as one of the most common malignant cancers, attracted the attention of many researchers. The main causes of HCC include infectious factors (like virus or parasite), and non-infectious factors (like alcohol, metabolic disorders, or aflatoxin). Recently, metabolic syndrome and its hepatic manifestation, nonalcoholic fatty liver disease, have been proved as a major cause of HCC. It was reported that cholesterol could contribute to lipotoxicity, inflammation, and fibrosis by reducing its exportation and excretion in HCC, however, higher cholesterol level might indicate a better disease-free and overall survival for HCC patients. Therefore, Zhou et al. reviewed and discussed contradictory roles of cholesterol in HCC, aiming to evaluate the potential of cholesterol as a therapeutic target (Zhou and Sun). Besides, the incidence of HCC is associated with stressful syndrome and relevant liver toxicity, such as cholestasis. Wu et al. demonstrated that arbutin exerts a protective effect on α-naphthylisothiocyanate-induced cholestasis liver toxicity *via* increasing levels of farnesoid X receptor and downstream enzymes that associate with bile acid metabolism.

Concerning treatments of HCC, Yan et al. indicated that under hypoxia autophagy-induced activation of histone deacetylase 6 (HDAC6) does not only promote the nuclear translocation of β-catenin, but also increases the interaction between β-catenin and the transcription repressor chicken ovalbumin upstream promoter-transcription factor 2. As a result, HDAC6 suppresses mitochondrial oxidative phosphorylation-related genes transcription and serves as a potential target to reduce the survival of HCC cells. Okuda et al. revealed that the combination of L-asparaginase and lenvatinib could synergistically inhibit proliferation and induce amino acid depletion-related oxidative stress, leading to the death of HCC cell lines, except Huh7 cells.

## Prospect and conclusion

As dysregulated stress response closely associates with cancer initiation and progression as well as the therapeutic effect of anti-cancer treatments, a better understanding of the dysregulated stress response during cancer development helps target key elements to achieve the anticancer purpose. The combination of different therapies could exert synergistic effects *via* modulating stress response signaling pathways. Besides, knowledges on the stress response could benefit the prediction of therapeutic efficacy and prognosis, which is critical for developing individualized treatment and follow-up management.

Although a lot of effort has been made to answer how cancer treatments modulate dysregulated stress response signaling to exert anticancer effects, the molecular mechanism underlying the connection between stress response and the regulation of therapeutic efficacy is still not clear. Cancer cells may develop resistance to therapies by different mechanisms, including patho-physiological, genetic, epigenetic, and micro-environmental changes in cancer cells during treatments (1–3). From a clinical point of view, it is important to further explore how cancer drug resistance associated with dysregulated stress response during cancer progression, so as to develop more effective therapeutic interventions. Also, more attention is needed to understand and balance benefits of treatment and related side effects of current cancer therapies.

In conclusion, this Research Topic aimed to discuss dysregulated stress response signaling pathways that are associated with cancer development and progression, as well as their implications for cancer treatments and post-treatment management.

## Author contributions

All authors listed have made a substantial, direct, and intellectual contribution to the work and approved it for publication.

## Funding

P.Z. is supported by CPRIT RR200089, NIH R00HL143277 and R01DK133304.

## Conflict of interest

The authors declare that the research was conducted in the absence of any commercial or financial relationships that could be construed as a potential conflict of interest.

## Publisher’s note

All claims expressed in this article are solely those of the authors and do not necessarily represent those of their affiliated organizations, or those of the publisher, the editors and the reviewers. Any product that may be evaluated in this article, or claim that may be made by its manufacturer, is not guaranteed or endorsed by the publisher.
